# Knowledge of Tunisian psychiatric residents in the field of sexology: A cross-sectional study

**DOI:** 10.1192/j.eurpsy.2026.12109

**Published:** 2026-07-31

**Authors:** S. Walha, K. Mahfoudh, R. Hosni, A. Ouertani, A. Aissa, R. Jomli

**Affiliations:** Psychiatric department “A”, RAZI Hospital, Mannouba, Tunisia

## Abstract

**Introduction:**

Sexuality refers to a wide range of experiences, thoughts, feelings, and behaviors related to sexual attraction, desire, expression, and identity. However, in Tunisia, patients’ sexual concerns are often underestimated due to the reluctance of some healthcare professionals to address sexual health issues. In this context, medical residents, particularly those specializing in psychiatry, are expected to soon be able to practice general medicine in Tunisia and provide appropriate care for their patients.

**Objectives:**

The objectives of our study were to assess the knowledge of Tunisian psychiatric residents in terms of sexuality, identify their sources of information, and determine the factors associated with their knowledge of patients’ sexuality.

**Methods:**

This was a descriptive and analytical study with cross-sectional data collection conducted on a population composed of a sample of psychiatry residents practicing in Tunisia at all levels: from the first to the fifth year. Our study was based on an online questionnaire and assessed participants’ knowledge of sexuality. It took place from March 1 to April 1, 2025.

**Results:**

Forty-two of the 90 psychiatrists approached (47%) agreed to participate in the study and completed the questionnaire. This resulted in a population of 42 young psychiatrists. The majority of psychiatrists participating in the study were female (83%; n=35) with a sex ratio of 5. The average age of these young practitioners was 27.95 years. The majority of participants (31%) were fourth-year psychiatry residents. More than half of the psychiatrists (55%; n=23) had received at least some training in sexology. Workshops and training seminars were the most common form of training (61%, n=27). With regard to knowledge about sexuality, the average percentage of correct answers was 70.73%. Among residents, 31% (N=13) scored 70.73% or higher on correct answers. Regarding factors influencing physicians’ knowledge, we found a significant relationship between the different stages of residency and pharmacological approaches to patients’ sexuality (p=0.001). Furthermore, a significant relationship was found between pharmacological approaches and residents with degrees in sexology (p=0.01). Finally, we found a significant relationship between the knowledge and attitudes of participating physicians toward patients’ sexuality (p=0.045).

**Conclusions:**

This study reveals several gaps in Tunisian doctors’ knowledge and perceptions regarding their patients’ sexuality. At the end of this study, we suggest that sexology be taught in all medical schools in Tunisia and, above all, that the acceptance capacity of residents in such studies be increased. This will promote the good sexual health of our patients and contribute to better mental balance. We also propose the creation of awareness programs for healthcare professionals.

**Disclosure of Interest:**

None Declared

